# Exploring the underlying structural mechanisms and whole-person perspectives on the desire for hastened death in patients with terminal cancer: A qualitative study

**DOI:** 10.1017/S1478951526102028

**Published:** 2026-04-07

**Authors:** Yuko Matsumura, Hiroki Kato, Eiko Maetaki, Kengo Imai, Yusuke Hiratsuka, Hideyuki Kashiwagi, Koji Amano, Yutaka Hatano, Masanori Mori, Tatsuya Morita, Yuki Shirai, Keiko Tamura, Keiko Sato

**Affiliations:** 1 Department of Advanced Nursing Sciences, School of Human Health Sciences, Graduate School of Medicine, Kyoto University, Kyoto, Japan; 2 Department of Nursing, Aino University, Osaka, Japan; 3 Faculty of Nursing, Graduate School of Nursing, Kansai Medical University, Osaka, Japan; 4 Department of Seirei Hospice, Seirei Mikatahara General Hospital, Shizuoka, Japan; 5 Department of Palliative Medicine, Takeda General Hospital, Aizuwakamatu, Japan; 6 Department of Transitional and Palliative Care, Iizuka Hospital, Fukuoka, Japan; 7 Department of Supportive and Palliative Care, Osaka International Cancer Institute, Osaka, Japan; 8 Department of Palliative Care, Yoshida Hospital, Nara, Japan; 9 Division of Palliative and Supportive Care, Seirei Mikatahara General Hospital, Shizuoka, Japan; 10 The Graduate School of Nursing Preparatory Office, Osaka Dental University, Osaka, Japan; 11 Department of Health Informatics, School of Public Health, Graduate School of Medicine, Kyoto University, Kyoto, Japan

**Keywords:** Terminal cancer, desire for hastened death, spiritual pain, suffering, whole-person approach

## Abstract

**Objectives:**

In this study, we aimed to elucidate the underlying structural mechanisms that generate a desire for hastened death (DHD) in patients with terminal cancer from a whole-person perspective based on insights from palliative-care professionals (PCPs).

**Methods:**

We conducted semi-structured interviews with 36 PCPs experienced in caring for patients with terminal cancer and DHD, followed by a thematic analysis based on Boyatzis’ hybrid approach.

**Results:**

We identified 6 themes that characterize the underlying structural mechanisms of DHD. DHD arises from feelings such as loss of self-control, inability to escape adverse circumstances, confronting death and letting go of life, pain of loneliness, being unable to accept living life as it is, and feeling unable to live with the thought of being an inconvenience to others, in addition to physical and psychological pain. In contrast, certain patients who had built good relationships with family members and/or PCPs found new meaning and value in their current lives, expressing a desire to live in the moment and choosing to continue living until the end.

**Significance of results:**

This study provides the first comprehensive analysis of the underlying structural mechanisms of DHD in patients with terminal cancer from a whole-person perspective. DHD with spiritual pain is linked to the loss of future orientation, autonomy, and meaningful relationships through interconnected structural pathways, leading to feelings of worthlessness and existential meaninglessness. The identified framework demonstrates that these underlying mechanisms operate through an interplay of existential, relational, and autonomy-related factors extending beyond physical and psychological symptoms, reflecting an interconnected human experience across physical, psychological, social, and spiritual dimensions. This study established an evidence-based framework enabling healthcare professionals to implement whole-person approaches to recognize the multidimensional nature of DHD and address existential distress across all dimensions of human experience in end-of-life care.

## Introduction

Patients with terminal cancer may express a desire for hastened death (DHD) (Chochinov et al. 1995; Morita et al. 2004b; Nissim et al. 2009; Monforte-Royo et al. 2011; Ferrand et al. 2012). In countries that recognize euthanasia or assisted dying, palliative-care professionals (PCPs) may assist in deaths of such patients (Monforte-Royo et al. 2011; Dierickx et al. 2018). In Japan, where euthanasia is not legalized, DHD is expressed as psychological or existential distress, rather than as a formal request (Morita et al. 2004b). According to a Delphi consensus study (Balaguer et al. 2016), DHD is defined as a response to suffering in the context of a life-threatening condition, where the patient sees no other option but to accelerate their death.

In contrast, the World Health Organization views death as a natural part of life, advocating for palliative-care approaches helping patients live as actively as possible until death (World Health Organization 2020). Many patients maintain their will to live until the end, finding meaning even while suffering from illness (Chochinov et al. 2002; Rodin et al. 2007; Rodríguez-Prat et al. 2017). However, healthcare workers often lack sufficient interpersonal support skills when encountering patients with DHD, resulting in feelings of helplessness, responsibility, and internal conflict (Ferrand et al. 2012; Galushko et al. 2016; Foo et al. 2021).

DHD is associated with physical, psychological, social, and spiritual pain and thus must be understood from the perspective of total pain (Morita et al. 2004b; Monforte-Royo et al. 2011; Balaguer et al. 2016). Moreover, identifying the type of suffering experienced by each patient is necessary to understand DHD (Cassell 1982; Balaguer et al. 2016; Rodríguez-Prat et al. 2017). Although DHD appears to arise from a complex interplay of these multidimensional forms of suffering, the underlying structural mechanisms through which they converge to generate DHD remain unclear.

Ohnsorge et al. (2014) categorized DHD causes into “reasons,” “meanings,” and “functions,” including physical (pain, loss of control), psychological (depression, anxiety), social (loss of social role, increased dependence), and spiritual pain (loss of meaning and goals in life). However, interrelationships among these factors and their contributions to DHD development remain unclear.

Pestinger et al. (2015) stated that patients with terminal cancer express DHD as a means of controlling distress linked to the dying process, highlighting that patients ultimately want healthcare workers to understand their suffering. However, the authors did not specify underlying structural mechanisms through which these relational factors influence DHD development, creating a knowledge gap that challenges healthcare professionals in providing effective care.

Furthermore, although measures are being developed to manage total pain causing DHD (Chochinov et al. 1995; Bellido-Pérez et al. 2017; Porta-Sales et al. 2019), understanding the types of pain that patients are less aware of, such as “loss of meaning,” remains challenging (Cassell 1999; Monforte-Royo et al. 2011; Ohnsorge et al. 2014; Rodríguez-Prat et al. 2017). The structures giving rise to DHD, including spiritual pain, remain unexplained (Hatano et al. 2021).

Murata and Morita (2006) defined the spiritual pain of patients with terminal cancer as “pain caused by extinction of the being and meaning of the self,” structured based on 3 components: temporality, existence within relationships, and existence with autonomy. Murata interpreted spiritual pain, including DHD, as arising from the combined loss of future orientation, personal autonomy, and relational connections (Murata 2003; Murata and Morita 2006). However, a comprehensive structural framework that elucidates the underlying structural mechanisms and explains how these elements interact to generate DHD has not yet been empirically established.

In this study, we define “underlying structural mechanisms” as the relational, cognitive, and contextual structures that organize and give rise to physical, psychological, social, and spiritual pain, ultimately generating DHD. This conceptual framework serves as an analytical lens to identify and interpret how multiple dimensions of suffering converge and interact to produce DHD in patients with terminal cancer.

Therefore, we aimed to assess total pain experienced by patients with terminal cancer from a whole-person perspective and to elucidate underlying structural mechanisms that produce DHD to provide an evidence-based framework for understanding how DHD develops in these patients, offering valuable insights for whole-person approach implementation in palliative-care settings.

## Methods

### Research design

A qualitative methodology using semi-structured interviews and thematic analysis (TA) was employed (Braun and Clarke 2006). TA is a qualitative analytical approach involving a systematic process identifying patterns within qualitative data. TA allows for a rich and nuanced understanding of participants’ experiences, making it particularly suitable for exploring complex phenomena such as DHD. The methodology and reporting adhered to the Consolidated Criteria for Reporting Qualitative Research (COREQ) (Tong et al. 2007) (Table S1).

### Setting

We targeted PCPs with firsthand experience in treating patients with terminal cancer who expressed DHD, as documented in the EASED (East-Asian cross-cultural collaborative Study to Elucidate the Dying process) study. This multicenter observational study was conducted in palliative-care units (PCUs) across Japan, South Korea, and Taiwan, aiming to explore realities of end-of-life care for patients with terminal cancer (Hatano et al. 2021). This secondary analysis included data from Japanese centers and additional interviews with PCPs caring of patients who expressed statements such as “I want to die soon” during PCU stays.

Reasons for DHD were collected based on the attending physician’s selection from 12 predefined categories reported previously (Chochinov et al. 1995; Morita et al. 2004b; Wilson et al. 2007) (Table S2).

### Selection of interviewees

Interviewees were selected using snowball sampling (O’Reilly and Parker 2012), wherein an EASED study representative initially selected 1 physician or nurse who met the participant-selection criteria, who subsequently nominated the next participant. This was to elicit in-depth information from real cases, enabling rich and detailed analysis. Although theoretical sampling and saturation are useful in grounded theory, we did not aim to sample until theoretical saturation was reached, as these concepts remain ambiguous in TA and lack valid assessment methods (O’Reilly and Parker 2012; Malterud et al. 2016).

Among 39 selected individuals, 2 declined to participate owing to time constraints and 1 owing to job relocation; 36 participants provided completed consent forms recording their age, occupation, and professional qualification. Interviews were conducted between June 22 and October 26, 2018, with no dropouts.

### Data collection

A semi-structured interview guide (Table S3) was developed through research-team discussions and was refined via pilot interview for face-validity assessment. Interviews were conducted in quiet, private workplaces. Questions designed to explore DHD complexity and variability were included, such as: “What distress caused the patient to express DHD?” and “How are the sources of distress that resulted in DHD related to one another?”

After a brief rapport-building conversation, we explained the study’s purpose and began the interview once questions were addressed. The first author (YM, female) and second researcher (HK, male) conducted face-to-face interviews of approximately 1 h each using an interview guide, with conversations recorded using a digital audio recorder. One person conducted the interview, while the other observed the interview process and took notes to assist with subsequent data interpretation. Audio recordings were transcribed verbatim by a professional transcription service and cross-checked for accuracy by the researchers. NVivo 12 was used for data organization (QSR International Pty Ltd 2018).

### Data analysis

A hybrid approach combining inductive and deductive analytical methods was employed (Boyatzis 1998). The hybrid approach first involves inductive coding, followed by theme reinterpretation using existing theoretical frameworks to enhance analytical rigor. In this study, we incorporated the concept of underlying structural mechanisms – defined as the relational, cognitive, and contextual structures that organize physical, psychological, social, and spiritual pain – as an analytic lens to guide the coding process and theme development. We adopted this concept to examine how various factors contributing to DHD are connected and interact to generate its underlying structural mechanisms.

Three researchers (YM, HK, and EM) coded the transcripts, which were recorded in a codebook using NVivo 12. The analysis commenced with repeated, thorough readings of verbatim transcripts and listening to audio recordings, ensuring familiarity with data before coding. Next, the researchers explored words and concepts deemed important regardless of theme and coded all verbatim transcripts sentence by sentence. Coding was based on the frequency of narrative repetition, intensity of emotion, and centrality of meaning within the overall narrative. Codes were arranged chronologically to explore participants’ perceptions of DHD-producing structures.

Categories were formed considering code commonalities and differences. Relationships between categories were examined among researchers for meaningfulness, clear definition, and representativeness. Disagreements were resolved through contextual re-examination and detailed transcript discussion.

Deductive methods based on Murata’s concept of spiritual pain concept (existence grounded in temporality, existence within relationships, and existence with autonomy) were used to examine category relationships and create a coding tree (Table S4). Comparative processes were repeatedly discussed, generating 6 typologized themes. Category and theme frequencies were quantified to support pattern identification while retaining qualitative depth.

### Reliability of analysis

A multidisciplinary team comprising oncology nursing faculty, a certified palliative-care physician, and a graduate student certified as oncology clinical nurse specialist conducted analysis. The first author coded all interviews and maintained a research journal for reflexivity. Two researchers (HK, EM) independently reviewed codes for reliability enhancement.

The team held 95 meetings between July 20, 2018, and March 28, 2024, discussing coding decisions and theme relationships. Code names, definitions, and analytical considerations were recorded in NVivo 12, developing a rigorous codebook. An audit trail verified the research process.

## Results

### Interviewee characteristics

Participants were selected from physicians or nurses with experience treating and caring for 82 patients with terminal cancer expressing DHD in 15 PCUs across Japan.

Participants included 36 PCPs, aged between 28 and 51 years, with semi-structured interviews lasting approximately 35–102 min/session (Table 1). Twenty-four participants held certifications from professional or academic organizations and had received special training to practice specialized palliative care for patients with cancer.

**Table 1.** Characteristics of participants

	Physician (*n* = 23)	Nurse (*n* = 13)	Total (*n* = 36)
Characteristics	*n* (%)		
Gender			
Female	3 (13.0)	12 (92.3)	15 (41.6)
Male	20 (86.9)	1 (7.6)	21 (58.3)
Age (years)			
<30	0	3 (23.0)	3 (8.3)
30–49	22 (95.6)	9 (69.2)	31 (86.1)
>50	1 (4.3)	1 (7.6)	2 (5.5)
Years of experience (years)			
<10	4 (17.3)	3 (23.0)	7 (19.4)
10–25	17 (73.9)	8 (61.5)	25 (69.4)
>25	2 (8.6)	2 (5.5)	4 (11.1)
Years of palliative care experience (years)			
1–5	8 (34.7)	8 (61.5)	16 (44.4)
6–10	9 (39.1)	2 (5.5)	11 (30.5)
>10	6 (26.0)	3 (23.0)	9 (25.0)
Specialty			
Certified specialist in palliative medicine	11 (47.8)	-	11 (30.5)
Certified specialist in cancer chemotherapy	3 (13.0)	-	3 (8.3)
Certified surgeon (surgical society)	4 (17.3)	-	4 (11.1)
Certified specialist in internal medicine	2 (8.6)	-	2 (5.5)
Certified pediatrician	2 (8.6)	-	2 (5.5)
Certified specialist in primary care	2 (5.5)	-	2 (5.5)
Certified palliative care nurse	-	4 (11.1)	4 (11.1)

Although interview durations varied, cases were selected based on the depth of the DHD-related narrative, ensuring that the narrative richness did not excessively differ between cases. Case selection and analytical weights were adjusted according to data depth and content richness.

### Results analysis

In this study, we positioned the concept of underlying structural mechanisms as the central analytical lens. Participants’ narratives revealed the structural mechanisms through which multiple dimensions of suffering interacted to generate DHD. This analytical perspective guided the development of the 6 themes presented below.

The TA of DHD-generating structures resulted in the following 6 themes derived from 802 codes: (1) loss of self-control and feeling unable to escape adverse circumstance; (2) facing death and letting go of life; (3) pain of loneliness; (4) feeling unable to live with the thought of being an inconvenience to others; (5) being unable to accept living life as it is; and (6) wanting to live in the moment. These themes are underlined throughout this article. Five themes were associated with total pain contributing to DHD, whereas 1 reflected a change in patients’ feelings from DHD to a desire to live.

To enhance thematic objectivity, we constructed a coding tree through deductive analysis of 47 categories based on Murata’s spiritual pain framework (Table S4). The number of responses corresponding to each category was calculated as a percentage of 82 patients with terminal cancer to increase pattern objectivity within these themes, and the codebook was reconstructed (Table S5).

Themes, representative categories, and illustrative participant quotes are presented in Table 2 and are detailed hereafter.

**Table 2.** Themes, categories, and Representative Participant Quotes

Themes and categories	Quote
Theme1. Loss of self-control and feeling unable to escape adverse circumstances	
1.1. Overlapping psychological and physical pain, with loss of emotional control due to suffering	One participant stated the following regarding a patient unable to control his emotions due to suffering as their psychological and physical pain increased:
	*“As well as physical problems, the patient may have felt unable to relax and developed obsessive-compulsive symptoms. However, I think this could also be related to a loss of self-sufficiency, so mood swings and related pain could also have had an effect.” (i_08_46)*
1.2. Long-term unbearable pain causing feelings of discouragement about the future	One participant stated the following regarding a patient who experienced unbearable pain, lost any hope of improving, felt unable to escape those painful circumstances, and lost any meaning in life:
	*“He felt bad from having a tube permanently inserted in his nose, was physically weak, and must have been in a lot of pain. Also, as the suffering continued and there were absolutely no signs of improvement, he probably lost any hope of recovering.” (i_12_71)*
1.3. Recalling painful memories; finding no meaning in continuing to live	One participant mentioned a patient who recalled earlier suffering, lost hope for the future, and felt resigned.
	*“The patient remembered suffering before. Yes, in a detached tone of voice, the patient said, ‘People can live because there are things they want to do, but I don’t feel like doing anything anymore. Life is meaningless.” (i_02_745)*
Theme 2. Facing death and letting go of life	
2.1. Feeling empty due to being unable to see a future, taking a resigned approach	One participant described how being confronted with the inability to see any way forward and losing their future caused patients to experience futility, assuming a resigned “who cares” stance, and expressing DHD:
	*“I think they went from feeling very hopeful about being cured and getting better to saying ‘I won’t get better,’ ‘I can’t live at home anymore,’ ‘there’s nothing left,’ feeling a bit resigned. So, rather than clear expressions like ‘I want to die soon,’ it was more like a ‘who cares’ kind of feeling.” (i_19_30)*
2.2. Feeling relieved by one’s impending death and letting go of life	One participant stated the following about a patient who felt relieved by being freed from the suffering of responsibilities and burdens, relinquishing the will to live:
	*“In some ways, this patient may have felt relieved because the end was in sight. He would be able to escape after having carried those burdens and responsibilities for so many years, and wouldn’t have to keep carrying them anymore. On the other hand, perhaps the patient no longer felt strongly about continuing to live on. Maybe making that decision let him feel at ease.” (i_26_09)*
2.3. Having no regrets and therefore strongly desiring not to live with remorse	One participant discussed an older adult who had no regrets about life and was satisfied with having lived it to the fullest, giving up on living to avoid remorse, and expressing DHD:
	*“She thought, ‘I don’t have to keep trying so hard,’ gave up instead, and let go. That’s based on the premise of always having worked hard until then. That’s why the patient wasn’t perturbed by reaching the end, and decided that she had ‘lived life to the full.’” (i_27_26)*
2.4. Wanting to pass on to the “other side” and reunite with family members	Several participants mentioned the following about patients aware of the future that will continue after their deaths, accepting their deaths, and stating that they wanted to pass on to the “other side” to meet deceased family members after escaping their painful circumstances:
	*“She said, ‘I’ll be able to meet my late husband,’ several times. I think she meant that she was ready for the journey and wanted her husband to come to meet her. I think that because she believed that kind of world existed after death, she was able to set off on that journey with peace of mind.” (i_17_07)*
Theme 3. Pain of loneliness	
3.1. Feeling anxious about dying alone	Several participants stated the following about patients who were aware that they were dying and felt afraid of dying alone, feeling isolated, asking not to be left alone, and expressing DHD to escape that feeling:
	*“He was afraid of being alone and asked his wife to ‘stay by his side.’ That is, his fear of death actually led him to want to end things. It’s painful to keep feeling that fear for a long time, so even if patients try to resist it at first, once they understand that it’s impossible, they start wanting to die quickly instead.” (i_21_23)*
3.2. Feeling sad that no one understands what is important	One participant discussed a patient who had lost any purpose in life due to his illness and expressed DHD due to suffering from feeling that no one understood what mattered to him or her:
	*“The patient was a karaoke teacher who loved singing, who kept on saying, ‘I can’t even sing out loud anymore. There’s no way anyone can understand how I’m suffering.’ Because of that, the patient felt like he had been left behind alone, and seemed very sad and lonely.” (i_03_736)*
3.3. Life becoming meaningless due to increasing loneliness from broken family bonds	Similarly, one participant discussed a patient who had lost family relationships that had given his life meaning, feeling lonely, and losing any goals in life:
	*“It felt like blood relationships were extremely meaningful to that patient. He kept asking, ‘call my daughter,’ and even asking for euthanasia, and when he realized the day before that he might not be able to see his daughter, that may have suddenly destroyed his goal of living a long life. The patient was really discouraged, too..” (i_31_15)*
Theme 4. Feeling unable to live with the thought of being an inconvenience to others	
4.1. Causing difficulties for family members, feeling worthless, and losing hope about life	One participant described an older adult who felt that “my existence is an inconvenience for my family; my life isn’t worth causing difficulties for my family.”
	*“After retirement age, when the patient was looking forward to the future, she fell ill, and there was no one left to take care of the family. The patient didn’t want to be an inconvenience for her daughters, so she asked for euthanasia.” (i_05_748)*
4.2. Feeling anxious and unsure about whether one should live, considering the future of the family	One participant mentioned a patient who was worried about his family’s future, feeling conflicted between “the pain of being a burden on my family” and “wanting to live for my family’s sake.”
	*“This patient was particularly worried about his young daughter, and spoke about having ‘worked hard to raise her.’ The patient was very worried about his daughter’s future, and seemed to be very distressed about leaving her behind and making her feel sad. The patient was conflicted, feeling, ‘I’m in so much pain that I want to die quickly, but I still want to live for her.’” (i_01_238)*
4.3. Losing the will to live from losing one’s purpose in life and being isolated from seken (various groups that a person is perceived as being part of)	Patients who had previously found meaning in life through contributing to society felt that they had been left behind and were worthless owing to their inability to fulfill their roles in society.
	*“There was one patient who was very passionate about his work and had been devoted to his own patients. Even after learning of the disease, this patient apparently kept working for some time. I think he felt pride in being a medical practitioner. However, once the patient had to stop working and lost any connections with seken, he lost any purpose in life, and felt worthless and that his existence was meaningless.” (i_07_91)*
4.4. No value in living as a disabled person while burdening others	One participant stated the following regarding a completely blind patient dependent on healthcare workers who felt that his life was not worth the effort:
	*“It seemed like that patient basically felt worthless. He was disabled and seemed to have spent his entire life feeling like an inconvenience or a burden on other people. He was probably always thinking, ‘there’s no point in a long life for people like me.’” (i_26_09)*
Theme 5. Being unable to accept living life as it is	
5.1. No value in living with shame	One participant stated the following about a patient feeling ashamed of being helped to use the toilet by healthcare workers, who struggled to accept living life as it was:
	*“This patient was originally very self-reliant, and it became difficult to maintain her self-esteem. I remember the patient saying, ‘I hate not being able to use the toilet by myself. My life is meaningless.” (i_11_75)*
5.2. Feeling deeply discouraged about living with such a pathetic appearance	One participant mentioned a patient expressing sadness about changes in her physical appearance, stating that “there’s no value in living like this.”
	*“This patient was very beautiful and paid a lot of attention to her appearance. So one day, she suddenly noticed it. Her body felt different, or her hands were swollen like balloons. The patient may have been too scared to look or touch, and couldn’t face it.” (i_03_442)*
5.3. Feeling worthless and tired of life	One participant stated the following about a patient who felt that life was meaningless if he or she could not have the lifestyle he or she wanted:
	*“She didn’t want to die because of the pain, but having no end in sight was painful in itself, and the patient felt that she had ‘already lived enough.’ For that patient, life meant shopping and enjoying plays, but her condition meant that these activities were impossible, and she clearly felt that she would never feel any happier.” (i_26_66)*
Theme 6. Wanting to live in the moment	
6.1. Increasing one’s will to live through self-encouragement and making an effort	This participant noted that the patient accepted being unable to move the way he or she wanted to, set goals of “slight improvement through rehabilitation,” and was thus able to move beyond DHD, regaining the will to live.
	*“There was no particular reason, but it was important that the patient felt it was worth doing. He made an effort to ‘try to train my upper body a little,’ and gradually became more optimistic. I don’t think there was any dramatic change based on something that someone said, but through the support that the patient received, he gradually came to accept things and started feeling the ‘need to do something.’ That’s how this patient found some meaning in life.” (i_09_44)*
6.2. Imagining “home” as the place where one belongs, living in the moment	Several participants stated the following about patients being aware of the possibility of going “home” to “their familiar dwelling” where they belonged, which became a source of hope and a goal in their lives:
	*“Apparently, a home nurse told him, ‘You might be able to go home.’ After that, I started hearing the patient say, ‘I want to live,’ and he set a final goal of living at home with his wife, which led to him feeling hopeful about life.” (i_28_20)*
6.3. Finding hope and meaning in life through family bonds	One participant mentioned the following about a patient who placed a lot of importance on family relationships, who found a purpose in life from hoping to *“make memories,”* giving her life meaning, and restoring her will to live:
	*“Hearing the patient say, ‘I want to die,’ and not being able to respond was painful for the family as well. However, her husband said that he ‘wanted to make a ring’ for their 10th wedding anniversary, so she made the effort to go out, and they spent time together deciding on the ring’s design and so on. The patient didn’t want her husband to see her looking weak and tried to hide her pain as much as possible. Even so, I was amazed by her strength in trying to keep hoping.” (i_05_702)*
6.4. Feeling grateful for healthcare workers’ thorough care and trusting them with confidence	One participant mentioned the following about a patient who was suffering but felt grateful for healthcare workers’ thorough care and entrusted everything to those workers, changing her state of mind to accepting the worsening disease:
	*“The patient said, ‘I’m grateful to the people I’ve met here.’ In particular, I think she felt greatly supported by the nurses who worked hard every day to help her feel even a little better and provided thorough care no matter how long it took. Rather than steadily feeling worse and strongly desiring a quick end, she accepted her current situation and seemed to naturally surrender or entrust things to others.” (i_17_07)*

#### Theme 1: Loss of self-control and feeling unable to escape adverse circumstances

Participants’ interviews clearly illustrated their experiences with patients with terminal cancer who expressed DHD due to loss of self-control and feeling unable to escape adverse circumstances. Participants stated that feelings of physical/psychological distress, helplessness, and awareness of being unable to escape painful circumstances produced hopelessness and loss of meaning in life, resulting in DHD.

Participants identified diverse physical symptoms, such as pain, dyspnea, fatigue, and lack of appetite, as reasons patients were unable to escape their painful circumstances. They mentioned the following:

- individuals unable to endure uncontrollable pain and writhing in agony,
- individuals discouraged about the future due to prolonged, unbearable pain, and
- individuals discouraged by their inability to eat, leading to loss of meaning in life, etc.

This suffering was described as persistent over time. Participants explained how patients were unable to envision a future, became deeply depressed, wondered “why I have to suffer like this,” and expressed DHD. Similar experiences were described in cases involving pain unresponsive to narcotics and respiratory distress. Some patients lost self-control due to unbearable pain, felt helplessness as they “couldn’t hang on any longer,” and expressed DHD.

#### Theme 2: Facing death and letting go of life

Participants reported that certain patients with terminal cancer expressed DHD by facing death and letting go of life. The decision to accept death was based on various factors, with some patients appearing resigned and indifferent regarding their impending death; some wanting things to end and feeling like they had lived enough; others wishing to reunite with deceased ones in the afterlife; and some believing their death was inevitable, seeking to organize their affairs and die peacefully.

Participants reported that for some patients, DHD was caused by an awareness of having no future, resulting in patients giving up on life, feeling helpless, and desiring a peaceful death.

#### Theme 3: Pain of loneliness

Participants acknowledged that DHD may arise from the pain of loneliness, caused by fears of dying alone, other people not understanding what matters to the patient, and feeling isolated owing to breaking off family relationships, among others.

In particular, they mentioned that feeling that no one understands their suffering leads patients to believe that they have no relationships with others, a main cause of feeling isolated.

#### Theme 4: Feeling unable to live with the thought of being an inconvenience to others

Participants clearly expressed DHD as feeling unable to live with the thought of being an inconvenience to others. They recognized DHD as arising from remorse about not fulfilling their roles and burdening others by relying on their care, feeling that their lives lacked sufficient meaning or value to deserve such effort, and feeling that if their continued living caused inconvenience, it would be better for their families if they did not exist.

Participants stated that patients regretted burdening their families with medical fees and care, expressing DHD because they felt guilty that “continuing to live is a waste of money.”

Some patients expressed DHD due to self-loathing based on dying and leaving their family without the support they need, saddening, or burdening their family.

#### Theme 5: Being unable to accept living life as it is

Participants recognized that DHD can arise from the inability to be one’s former self or being unable to accept living life as it is, losing the ability to enjoy pleasurable experiences, and feeling that living presents no value or meaning.

The most common report concerned patients feeling despair over the loss of their sense of self due to a lack of independence. This was followed by hopelessness from having to depend on others for care as their disease progressed.

Patients experienced severe depression and despair regarding irreversible changes in appearance caused by their disease, leading to DHD.

Among older adults, some expressed DHD based on the feeling that their lives were worthless because their illness prevented them from enjoying pleasurable activities.

#### Theme 6: Wanting to live in the moment

Participants reported that although their patients expressed DHD, some simultaneously expressed the will to live, mentioning feelings of wanting to live in the moment. Their statements clarified that some patients accepted the situation and tried to find things they could do, finding meaning and goals in life.

One participant mentioned a patient who underwent a process of self-encouragement and made efforts to find value in living with his current condition. Participants recognized that among patients who expressed DHD, some could be encouraged to redefine themselves by accepting their suffering, building supportive relationships with healthcare workers and family members, and accepting any changes in themselves through self-reflection. Additionally, they emphasized that patients could perceive value in their lives through such processes.

## Discussion

Our results highlight the complex nature of suffering and the will to live among terminally ill patients with cancer. This discussion examines the following: (1) the underlying structural mechanisms characterizing DHD and (2) whole-person approaches that enable meaningful living until the end analyzed from a whole-person perspective based on 6 extracted themes.

### Underlying structural mechanisms of DHD-generating structures

This analysis draws on Murata’s theory of human existence, which defines spiritual pain as “pain caused by extinction of the being and the meaning of the self” across dimensions of temporality, relationships, and autonomy (Murata 2003; Murata and Morita 2006) (Table S4).

Experiencing loss of self-control and feeling unable to escape adverse circumstances are major causes of DHD in patients with physical and psychological distress. This overlaps with helplessness, creating a structure resulting in severe hopelessness and loss of meaning. Cassell (1982) defines suffering as a human reaction to feeling threatened by the loss of identity or valued way of life, noting that patients perceive death as the absence of future, leading to lost goals and meaning. Murata and Morita (2006) identified suffering as produced by divergence between objective situation and subjective hopes and values.

PCPs identified that patients experienced loss of self-control due to unbearable pain, perceived absence of hope for alleviation, feeling unable to return to former selves, and loss of meaning in life. Symptoms, including respiratory distress, fatigue, and depression, greatly reduced quality of life, with pharmacological intervention often proving challenging. When patients realized they could not escape suffering, they began questioning why they should continue, resulting in strong DHD tendencies.

This perception closely resembles Galynker’s (2023) “suicide crisis syndrome,” where individuals experiencing inescapable, unbearable pain are at high risk of fatal suicidal behaviors. Galynker emphasizes that healthcare providers should focus on understanding whether patients feel trapped rather than asking about suicidal intent. Japan’s General Principles of Suicide Prevention Policy (Ministry of Health, Labour and Welfare 2023) states that suicide should be regarded not as an instantaneous action but as a process of being driven into feeling no choice but to end one’s life.

Healthcare workers play critical roles in such situations. Accurate recognition of suffering is crucial (Cassell 1982, 1999; Balaguer et al. 2016). Appropriate interventions include medical support through medication adjustments, enhanced psychological support (Morita et al. 2004a; Hatano et al. 2021), and informing patients about the availability of palliative options, including sedatives (Imai et al. 2020; Hatano et al. 2021). Helping patients find goals and meaning through supportive communication and fostering trusting relationships are equally important (Cassell 1999; Murata 2003). Such diverse approaches can aid patients in recognizing alternatives to death, preventing processes leading to DHD. However, further research is needed to clarify these diverse approaches and their effectiveness in clinical practice.

For some patients, facing a terminal prognosis renders the present meaningless, representing spiritual pain related to temporality (Murata 2003; Murata and Morita 2006). Heidegger (1997) describes human life as a temporal structure where the present exists with support from the future and historicality. The prospect of a future may drive meaning in the present, while in other cases meaning is derived from accepting the absence of a future.

PCPs observed 3 patterns in patients’ attitudes toward death: feeling they had “lived enough” (particularly older adults), losing hope and feeling resigned, or desiring peaceful death but feeling discouraged about its possibility.

Some Western cultures accept DHD as an “autonomous choice” or “safety net,” considering it part of life (Nissim et al. 2009; Ohnsorge et al. 2014; Rodríguez-Prat et al. 2017, 2021; Dierickx et al. 2018). DHD may not simply reflect rejection of life but rather deciding how to conduct one’s life; however, in some cases, depression and poorly controlled symptoms causing despair may also contribute to DHD (Chochinov et al. 1995; Breitbart et al. 2000; Monforte-Royo et al. 2011; Hatano et al. 2021). For resigned patients, establishing environments for living “in the moment” is important, directing attention toward the near future (Chochinov et al. 2002; Murata 2003; Mount et al. 2007).

In Japan, individuals exist within a distinctive cultural construct known as “seken” (Abe 1995; Iwamoto 2021) – a complex relational framework encompassing family, workplace, and social groups transcending simple group memberships to form an interconnected social matrix. Each individual is perceived as part of several interwoven societies and exists within their meaningful connections with others, i.e., “has a place in seken” (Abe 1995). Individual value and life meaning arise from these relational bonds within seken; loss of such ties causes profound futility and distress (Boston and Mount 2006; Murata and Morita 2006; Best et al. 2015).

PCPs identified 3 patterns of loneliness: fear of dying alone and separation from family and lived world; loss of purpose when unable to share suffering with those who understood their identity; and impossibility of repairing important relationships, preventing reconnection with community. This loneliness represents not merely psychological problems but instead suffering from losing one’s place in seken, disrupting existence, and causing greater spiritual pain (Murata 2003; Murata and Morita 2006).

When patients feel that no one understands their suffering, they experience loss of the very basis of their existence. Therefore, understanding and showing empathy with suffering can support patients’ feeling reconnected with seken, feeling that their lives are worthy, and regaining purpose.

The value of “not being an inconvenience” is internalized within seken’s communal framework (Abe 1995; Iwamoto 2021), leading to autonomy loss and relationship distancing. Individuals are required to avoid causing friction and maintain harmony (Abe 1995; Iwamoto 2021), explaining why patients feel unable to live when dependent on others. Additionally the inability to maintain self-image and the necessity of exposing private aspects greatly influences self-respect and relationships, leading to dignity and self-worth loss (Best et al. 2015; Rodríguez-Prat et al. 2019, 2021; Borges et al. 2024).

Although Western societies discuss this in “right to die” contexts rooted in liberal individualism (Dierickx et al. 2018; Veatch and Guidry-Grimes 2019; Rodríguez-Prat et al. 2021; Borges et al. 2024), the Japanese “intimacy-oriented culture” involves networks of dependencies wherein relationships heavily influence self (Kasulis 2002). Watsuji (2007) argues that “humanity is formed between individual agency and social harmony.” Accordingly, DHD in Japanese society is not simply an individual choice but a phenomenon arising from patients’ deep awareness of their relationships with their family and community. This cultural context means that DHD stems from anxiety about community acceptance and self-doubt regarding the value of living while inconveniencing others.

Therefore, following a structural assessment of spiritual pain, considering existence with autonomy and existence within relationships is essential to understanding the underlying structural mechanisms that lead to DHD. Moreover, improving our understanding of the culture and value-based foundations of DHD is crucial for investigating strategies for managing patients’ suffering from a whole-person perspective.

### Whole-person perspective: Enabling meaningful living until the end

While wanting to live in the moment can reflect the will to recover, in terminally ill patients, it often reflects finding meaning and purpose in the present despite approaching death. Through mutual trust relationships with PCPs and families, patients can set new goals and redefine themselves (Table S4).

Dying involves continuous loss experiences – role, societal place, existence itself (Kellehear 2009; Best et al. 2015). This extremely subjective, multilayered suffering proves difficult for healthcare workers to grasp through external symptom observation (Cassell 1999; Kellehear 2009).

Healthcare workers should approach such ever-changing suffering by showing empathy and attempting to understand patients’ lived experiences from an internal perspective. Washida (2001) argued that “listening” means accepting and acknowledging patient suffering, enabling patients to calmly consider themselves. The power of listening lies in being unconditionally present within others’ existence, forming foundations for patient support. This concept aligns closely with Chochinov’s notion of “Intensive Caring,” which emphasizes non-abandonment and a full presence with patients, communicating that they matter (Chochinov 2023).

**Figure 1.**
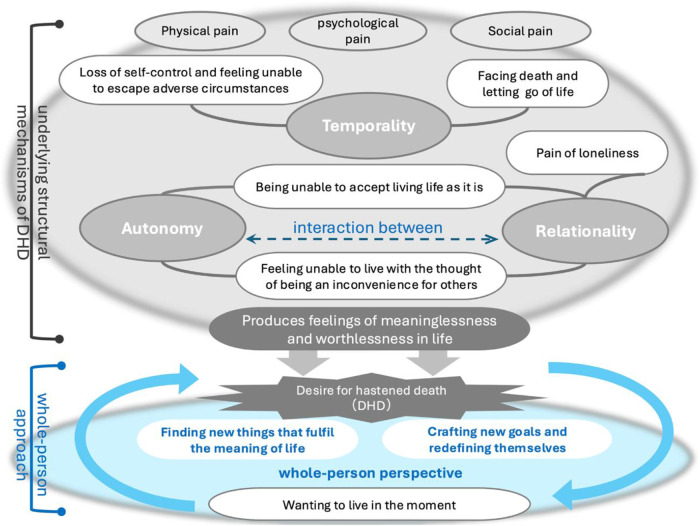
Conceptual framework for evaluating desire for hastened death (DHD).

Figure 1 illustrates the structural DHD evaluation framework from a whole-person perspective, demonstrating the underlying structural mechanisms through which comprehensive relational support addresses patients’ multidimensional needs to provide meaning in life, encourage self-redefinition, and restore their will to live, enabling meaningful living until the end.

Understanding patients’ worldviews from multidimensional perspectives is necessary for managing DHD. Support rooted in relationships represents a whole-person approach, enabling comprehensive support for patients’ lives and lifestyles, providing meaning, encouraging self-redefinition, and restoring the will to live.

This structural analysis contributes to end-of-life care by revealing DHD’s complex etiology beyond physical and psychological symptoms, emphasizing the need for culturally-informed, relationship-based interventions addressing existential distress’s multidimensional nature.

### Study strengths and limitations

This study clarifies the underlying structural mechanisms through which inescapable suffering contributes to DHD expression, emphasizing how the loss of the future, autonomy, and relationships operates through interconnected structural pathways, leading to diminished meaning in life. It highlights how PCPs’ whole-person perspective can help patients rediscover meaning and regain the will to live, offering practical implications for care. These insights were drawn from comprehensive interviews with experienced PCPs reflecting on patient conditions and treatment processes.

However, the study had several limitations. First, given that it relied on PCPs’ perspectives, patients’ inner experiences were indirectly captured. Second, older adults were over-represented, limiting generalizability to adolescents, young adults, and pediatric patients. Third, findings were based on PCU experiences, with uncertain generalizability to general wards, and patients without DHD were excluded, restricting understanding of preventive approaches.

This research contributes to end-of-life care by emphasizing whole-person approaches addressing existential distress’s multidimensional nature, enabling meaningful living until death. However, future research should encompass diverse age groups, settings, and social backgrounds to validate findings, explore relationships between insights and patients’ peaceful death desires, and develop evidence-based supportive measures.

## Supporting information

10.1017/S1478951526102028.sm001 Matsumura et al. supplementary material 1 Matsumura et al. supplementary material

10.1017/S1478951526102028.sm002 Matsumura et al. supplementary material 2 Matsumura et al. supplementary material

10.1017/S1478951526102028.sm003 Matsumura et al. supplementary material 3 Matsumura et al. supplementary material

10.1017/S1478951526102028.sm004 Matsumura et al. supplementary material 4 Matsumura et al. supplementary material

10.1017/S1478951526102028.sm005 Matsumura et al. supplementary material 5 Matsumura et al. supplementary material

## Supplementary material

The supplementary material for this article can be found at https://doi.org/10.1017/S1478951526102028.
