# Supplementary material for: Exploring the underlying structural mechanisms and whole-person perspectives on the desire for hastened death in patients with terminal cancer: A qualitative study
Source: Palliat Support Care. 2026 Apr 7;24:e100. doi: 10.1017/S1478951526102028 (PMC13166461; doi:10.1017/S1478951526102028)
Supplement: Matsumura et al. supplementary material 1 — Matsumura et al. supplementary material [file S1478951526102028sup001.docx]

# ***Supplementtable1: Consolidated Criteria for Reporting Qualitative Research (COREQ)***

| No | Item | Description | Answer | Appears in Section |
| --- | --- | --- | --- | --- |
| **Domain 1: Research team and reflexivity**  **Personal characteristics** | | | | |
| 1 | Interviewer/facilitator | Who conducted the interviews? | YM (first author) and HK (second researcher) | 2 Method  2.4 Data collection |
| 2 | Credentials | What are the researchers' qualifications? | YM, HK=PhD student, EM=Nurse | Not applicable |
| 3 | Occupation | What was their occupation at the time of the study? | Nurse | Not applicable |
| 4 | Gender | Were the researchers male or female? | YM, EM=female, HK=male | 2 Method  2.4 Data collection |
| 5 | Experience and training | What experience or training did the researchers have? | We formed a research team that included researchers with extensive experience in interviews and qualitative research. | 2 Method  2.6 Reliability of Analysis |
| **Relationship with participants** | | | | |
| 6 | Relationship established | Was a relationship established prior to study commencement? | Before beginning the interviews, a casual conversation was incorporated for rapport building (establishing trust relationships) with participants. | 2 Method  2.4 Data collection |
| 7 | Participant knowledge of the interviewer | What did the participants know about the researcher? | The researchers explained the purpose of this study and that it was incorporated into their doctoral dissertation research theme. | 2 Method  2.4 Data collection |
| 8 | Interviewer characteristics | What characteristics were reported about the interviewer? | The interviewers were graduate students with specialized knowledge in palliative care, and some held certification as oncology nurse specialists. | 2 Method  2.6 Reliability of Analysis |
| **Domain 2: Study design Theoretical framework** | | | | |
| 9 | Methodological orientation and theory | Was the methodological orientation that supported the study indicated? | We employed thematic analysis (TA) as proposed by Boyatzis, which is a systematic process for identifying patterns within qualitative data. | 2 Method  2.1 Research Design |
| **Participant selection** | | | | |
| 10 | Sampling | How were participants selected? | Participants were selected using the snowball sampling method. | 2 Method  2.3 Selection of interviewees |
| 11 | Method of approach | How were participants approached? | We initially selected one physician or nurse who met the participant-selection criteria, who subsequently nominated the next participant. | 2 Method  2.3 Selection of interviewees |
| 12 | Sample size | How many people took part in the study? | Thirty-six participants. | 2 Method  2.3 Selection of interviewees |
| 13 | Non-participation | How many people refused to participate or dropped out? What were the reasons? | Three individuals declined participation, citing busy work schedules and workplace relocations as reasons. No participants dropped out once enrolled in the study. | 2 Method  2.3 Selection of interviewees |
| **Setting** | | | | |
| 14 | Setting of data collection | Where were the data collected? | Data were collected at the participants’ workplaces in a quiet setting comprising private rooms that ensured privacy. | 2 Method  2.4 Data collection |
| 15 | Presence of non-participants | Were there any individuals present besides the participants and the researchers? | Only HK, the second researcher, was present. | 2 Method  2.4 Data collection |
| 16 | Description of sample | What were the key characteristics of the sample? | See Table 1. | Table1 |
| **Data collection** | | | | |
| 17 | Interview guide | Were questions, prompts, or guides provided by the authors? Was a pilot test conducted? | See Supplementtable3.  We conducted preliminary interviews to test face validity. | 2 Method  2.4 Data collection  Supplementtable3 |
| 18 | Repeat interviews | Were repeat interviews conducted? If so, how many? | No: Repeat interviews were not conducted. | Not applicable |
| 19 | Audio/visual recording | Were audio or video recordings used for data collection? | Conversations were recorded using a digital audio recorder. | 2 Method  2.4 Data collection |
| 20 | Field notes | Were field notes made during and/or after the interviews? | During the interviews, the second researcher made observational records of the interview process to assist in data interpretation. | 2 Method  2.4 Data collection |
| 21 | Duration | What was the duration of the interviews? | Approximately 35-102 minutes per session. | 3 Result  3.1 Interviewee Characteristics |
| 22 | Data saturation | Was data saturation discussed? | While data saturation is useful as an element of constant comparative analysis in Grounded Theory, in TA, we did not aim for saturation due to the ambiguity of the concept and the lack of valid assessment methods. | 2 Method  2.3 Selection of interviewees |
| 23 | Transcripts returned | Were transcripts returned to participants for comment and/or correction? | No: Member checking was not performed. | Not applicable |
| **Domain 3: Analysis and findings Data analysis** | | | | |
| 24 | Number of data coders | How many data items were coded? | Six themes were extracted from 802 codes across 47 categories. Category and theme frequencies were quantified to identify patterns while maintaining qualitative depth. | 3 Result  3.2 Results analysis  Supplementtable5 |
| 25 | Description of the coding tree | Did the authors provide a description of the coding tree? | Using a hybrid approach analytical method, we repeatedly compared similarities and differences in codes across participants, and these interpretations were discussed repeatedly by the entire research team. | 2 Method  2.5 Data Analysis  Supplementtable4 |
| 26 | Derivation of themes | Were themes identified a priori or derived from the data? | We first conducted inductive analysis, then deductively interpreted the six generated themes using the concept of spiritual pain. | 2 Method  2.5 Data Analysis  Supplementtable5 |
| 27 | Software | What software, if applicable, was used to manage the data? | NVivo 12 (qualitative research support software) | 2 Method  2.4 Data Collection  2.5 Data Analysis |
| 28 | Participant checking | Did participants provide feedback on the research findings? | No: Feedback was not obtained. | Not applicable |
| **Reporting** | | | | |
| 29 | Quotations presented | Were participant quotations presented to illustrate the themes/findings? Each quotation identified? (e.g., participant number) | Yes: Quotations were identified by participant number, terminal cancer patient number.  See Table 2. | Table 2 |
| 30 | Data and findings consistent | Was there consistency between the data presented and the findings? | Yes: The themes demonstrated internal consistency and were coherent and distinctive. | 3 Result  4 Discussion |
| 31 | Clarity of major themes | Are the main themes clearly presented in the findings? | Yes: Clearly presented | 3 Result  4 Discussion |
| 32 | Clarity of minor themes | Are diverse examples or discussions of sub-themes explained? | Yes: They are explained. | 3 Result  4 Discussion |
