# Supplementary material for: Exploring the underlying structural mechanisms and whole-person perspectives on the desire for hastened death in patients with terminal cancer: A qualitative study
Source: Palliat Support Care. 2026 Apr 7;24:e100. doi: 10.1017/S1478951526102028 (PMC13166461; doi:10.1017/S1478951526102028)
Supplement: Matsumura et al. supplementary material 2 — Matsumura et al. supplementary material [file S1478951526102028sup002.docx]

# ***Supplementtable2: Characteristics of Patients with Terminal Cancer Who Expressed DHD***

| Characteristics | N=82 | % |
| --- | --- | --- |
| Age (Mean; Range) | 70; 34–91 |  |
| Sex  Male  Female | 47  35 | 57.3  42.6 |
| Primary Cancer Site  Gastrointestinal  Liver/Biliary/Pancreas  Lung  Breast  Urogenital  Gynecological  Other | 25  12  10  7  7  3  18 | 30.4  14.6  12.1  8.5  8.5  3.6  21.9 |
| ECOG Performance Status  1  2  3  4 | 0  9  41  32 | 0  10.9  50  39 |
| Length of PCU Stay (days) (Mean; Range) | 33.5; 1–130 |  |
| Time of DHD Expression (days) (Mean; Range) | 11: 1–20 |  |
| Marital Status  Single  Married  Widowed  Divorced | 11  48  13  10 | 13.4  58.5  15.8  12.1 |
| Family Living Together  Yes  No | 61  21 | 74.3  25.6 |
| Religion  No specific religion  Buddhism  Other  Not known | 36  3  4  39 | 43.9  3.6  4.8  47.5 |
| Preferences and Decision-Making  Chosen PCU to avoid burdening others  No  Yes | 43  39 | 52.4  47.5 |
| Desire to hasten death (by definition)  Wish to hasten death  Request for death (desire for euthanasia) | 39  43 | 47.5  52.4 |
