# Supplementary material for: Exploring the underlying structural mechanisms and whole-person perspectives on the desire for hastened death in patients with terminal cancer: A qualitative study
Source: Palliat Support Care. 2026 Apr 7;24:e100. doi: 10.1017/S1478951526102028 (PMC13166461; doi:10.1017/S1478951526102028)
Supplement: Matsumura et al. supplementary material 3 — Matsumura et al. supplementary material [file S1478951526102028sup003.docx]

# ***Supplementtable3: Interview Guide***

| Topics | Questions asked |
| --- | --- |
| Presence of DHD | ・When and how was DHD expressed? |
| Complexity of DHD | ・What distress caused the patient to express DHD?  ・How are the sources of distress that resulted in DHD related to one another?  ・Was there a DHD expression that was not caused by distress? |
| Variability of DHD | ・Did the DHD expression change from stronger to weaker?  ・Did the DHD expression ever disappear? |
| Communality of DHD | ・Why do you think the patient expressed DHD? |
| Approaches to DHD | ・What palliative care was provided to patients with DHD? |
