# Supplementary material for: Exploring the underlying structural mechanisms and whole-person perspectives on the desire for hastened death in patients with terminal cancer: A qualitative study
Source: Palliat Support Care. 2026 Apr 7;24:e100. doi: 10.1017/S1478951526102028 (PMC13166461; doi:10.1017/S1478951526102028)
Supplement: Matsumura et al. supplementary material 4 — Matsumura et al. supplementary material [file S1478951526102028sup004.docx]

# ***Supplementtable4: Coding Tree***

# ***Theme 1: Loss of self-control and feeling unable to escape adverse circumstances***

| ***1. Loss of self-control and feeling unable to escape adverse circumstances*** | | |
| --- | --- | --- |
|  | ***1.1. Suffering due to the loss of future*** | |
|  |  | ・Recalling painful memories; feeling that life is meaningless |
|  |  | ・Inability to see a future and becoming deeply depressed |
|  |  | ・Fading consciousness, losing the ability to complain of pain |
|  |  | ・Being crushed by a feeling of helplessness |
|  |  | ・Feeling discouraged about the future due to unbearable pain |
|  |  | ・Loss of self-control |
|  |  | ・Causes preventing escape from suffering (pain, respiratory distress, fatigue, lack of appetite, etc.) |
|  |  | ・Loss of emotional control due to suffering |
|  |  | ・Suffering that cannot be expressed in words |
|  |  | ・Writhing in uncontrollable agony |
|  |  | ・Feeling discouraged by not being able to eat |

# ***Theme 2: Facing death and letting go of life***

| ***2. Facing death and letting go of life*** | | |
| --- | --- | --- |
|  | ***2.1. Suffering due to the loss of future*** | |
|  |  | ・Wanting to meet deceased people in the afterlife |
|  |  | ・Having lived enough and wanting it to end now |
|  |  | ・Desire to die peacefully |
|  |  | ・Not wanting to live with remorse |
|  |  | ・Awareness of the future that will continue after one’s death |
|  |  | ・Feeling tired of living and wanting to rest |
|  |  | ・Feeling resigned due to futility |
|  |  | ・Relief from freeing oneself of responsibilities and burdens |
|  |  | ・Wanting to die a clean death after organizing one’s affairs |

# ***Theme 3: The pain of loneliness***

| ***3. The pain of loneliness*** | | |
| --- | --- | --- |
|  | ***3.1. Suffering due to the loss of relationships with others*** | |
|  |  | ・Fear of dying alone |
|  |  | ・Being overcome by loneliness and rage |
|  |  | ・Thinking that life is meaningless when feeling lonely |
|  |  | ・Broken family ties, feeling very lonely |
|  |  | ・No one understands what is important |

# ***Theme 4: Feeling unable to live with the thought of being an inconvenience to others***

| ***4. Feeling unable to live with the thought of being an inconvenience to others*** | | |
| --- | --- | --- |
|  | ***4.1. Suffering due to the interactive loss of autonomy and relationships*** | |
|  |  | ・Inability to fulfill one’s seken role (various groups that a person is perceived as being part of) |
|  |  | ・Feeling guilty for burdening family members with medical expenses and care |
|  |  | ・Self-loathing due to causing sadness and burdens for family members |
|  |  | ・Causing difficulties for family members and having a worthless life |
|  |  | ・Feeling unsure whether one should live, considering the future of the family |
|  |  | ・Being a burden on one’s family and seken (various groups that a person is perceived as being part of) |
|  |  | ・It would be better not to exist for the family’s sake |
|  |  | ・Being alive is an inconvenience for other people |
|  |  | ・No value in depending on others to live as a disabled person |

# ***Theme 5: Being unable to accept living life as it is***

| ***5. Being unable to accept living life as it is*** | | |
| --- | --- | --- |
|  | ***5.1. Suffering due to the interactive loss of autonomy and relationships*** | |
|  |  | ・Inability to accept living like this |
|  |  | ・Living in this condition is meaningless |
|  |  | ・Feeling embarrassed to ask others for help using the toilet |
|  |  | ・Hopelessness from having to depend on others for care |
|  |  | ・Feeling deeply discouraged by changes in one’s physical appearance |
|  |  | ・Inability to enjoy pleasurable activities makes life worthless |
|  |  | ・Extending life through treatment is meaningless, and living is worthless |
|  |  | ・Inability to be one’s true self |
|  |  | ・Losing one’s sense of self due to a lack of independence |

# ***Theme 6: Wanting to live in the moment***

| ***6. Wanting to live in the moment*** | | |
| --- | --- | --- |
|  | ***6.1. Finding new things that bring meaning to life*** | |
|  |  | ・Finding hope and meaning in living through family bonds |
|  |  | ・Refraining from thoughts of dying due to a sense of family responsibilities |
|  |  | ・Imagining “home” as the place where one belongs |
|  |  | ・Self-encouragement and making an effort |
|  | ***6.2. Crafting new goals and redefining themselves*** | |
|  |  | ・Building relationships with healthcare workers, taking a new look at oneself |
|  |  | ・Feeling grateful for healthcare workers’ thorough care |
|  |  | ・Relaxing in a comfortable environment |
|  |  | ・Suppressing anxiety and uncertainties about death |
|  |  | ・Pain relief fosters emotional calm and positivity |
