# Supplementary material for: Exploring the underlying structural mechanisms and whole-person perspectives on the desire for hastened death in patients with terminal cancer: A qualitative study
Source: Palliat Support Care. 2026 Apr 7;24:e100. doi: 10.1017/S1478951526102028 (PMC13166461; doi:10.1017/S1478951526102028)
Supplement: Matsumura et al. supplementary material 5 — Matsumura et al. supplementary material [file S1478951526102028sup005.docx]

# ***Supplementtable5: Response Frequency by Theme Names and Categories (N=82)***

# ***Theme 1: Loss of self-control and feeling unable to escape adverse circumstances (N=78/95.1%)***

***(Response Frequency by Theme Names and Categories)***

| Categories | Number of patients who expressed DHD（N＝78/95.1%） | |
| --- | --- | --- |
|  | N | ％ |
| Fading consciousness leading to losing the ability to complain of pain | 34 | 41.4 |
| Overlapping psychological and physical pain, with loss of emotional control due to suffering | 29 | 35.3 |
| Feeling overcome by unbearable pain and a strong sense of helplessness | 27 | 32.9 |
| Uncontrollable pain and writhing in agony | 23 | 28.0 |
| Recalling painful memories; finding no meaning in continuing to live | 22 | 25.6 |
| Fading consciousness and suffering that cannot be expressed in words | 19 | 23.1 |
| Falling into the depths of despair and crying, “Why me?” | 15 | 18.2 |
| Long-term unbearable pain causing feelings of discouragement about the future | 15 | 18.2 |
| Severe pain ravaging the patient’s mind and body, being unable to resist | 12 | 14.6 |
| Feeling discouraged about the inability to eat, losing any meaning in life | 4 | 4.8 |

# ***Theme 2: Facing death and letting go of life (N=52/63.4%)***

***(Response Frequency by Theme Names and Categories)***

| Categories | Number of patients who expressed DHD（N＝52/63.4%） | |
| --- | --- | --- |
|  | N | ％ |
| Feeling empty due to being unable to see a future, taking a resigned approach | 20 | 25.6 |
| Having no regrets and therefore strongly desiring not to live with remorse | 14 | 17.0 |
| Calmly observing the approach of death and making a decision | 11 | 13.4 |
| Feeling relieved by one’s impending death and letting go of life | 11 | 13.4 |
| Making one’s own decisions about sedation and where to die in preparation for death | 11 | 13.4 |
| Preferring a graceful death to living while depending on others | 9 | 10.9 |
| Wanting to pass on to the “other side” and reunite with family members | 7 | 8.5 |
| Preparing for the funeral and body donation as proof of one’s life | 7 | 8.5 |
| Wanting to die a clean death after organizing one’s affairs | 5 | 6.0 |

# ***Theme 3: The pain of loneliness (N=47/57.3%)***

***(Response Frequency by Theme Names and Categories)***

| Categories | Number of patients who expressed DHD（N＝47/57.3%） | |
| --- | --- | --- |
|  | N | ％ |
| Feeling anxious about dying alone | 20 | 24.3 |
| Feeling sad that no one understands what is important | 20 | 24.3 |
| Overcome by a strong feeling of loneliness with the fear of impending death | 14 | 17.0 |
| Life becoming meaningless due to increasing loneliness from broken family bonds | 10 | 12.1 |
| Loneliness on feeling the approach of death, being overcome by rage | 9 | 10.9 |

# ***Theme 4: Feeling unable to live with the thought of being an inconvenience for others (N=49/59.7%)***

***(Response Frequency by Theme Names and Categories)***

| Categories | Number of patients who expressed DHD（N＝49/59.7%） | |
| --- | --- | --- |
|  | N | ％ |
| Suffering from feeling guilty about being an inconvenience for others, losing one’s meaning for existing | 17 | 20.7 |
| Burdening family with medical fees and care, feeling that one’s existence is worthless | 16 | 19.5 |
| Losing the will to live from losing one’s purpose in life and being isolated from seken | 14 | 17.0 |
| Feeling anxious and unsure about whether one should live, considering the future of the family | 12 | 14.6 |
| Causing difficulties for family members, feeling worthless, and losing hope about life | 11 | 13.4 |
| No value in living as a disabled person while burdening others | 6 | 7.3 |
| Feeling self-loathing due to both the sadness of one’s family and the sadness of burdening one’s family | 4 | 4.8 |

# ***Theme 5: Being unable to accept living life as it is (N=69/84.1%)***

***(Response Frequency by Theme Names and Categories)***

| Categories | Number of patients who expressed DHD（N＝69/84.1%） | |
| --- | --- | --- |
|  | N | ％ |
| Despairing about the situation and losing one’s sense of self due to a lack of independence | 25 | 30.4 |
| Feeling hopeless about one’s lack of independence and dependency on others | 22 | 26.8 |
| Feeling hopeless about being unable to live the way one wants, losing sight of the meaning of existing | 20 | 24.3 |
| No value in living with shame | 19 | 23.1 |
| Feeling worthless and tired of life | 19 | 23.1 |
| Treatment to extend one’s life is excessive and worthless | 18 | 21.9 |
| Feeling deeply discouraged about living with such a pathetic appearance | 11 | 13.4 |

# ***Theme 6: Wanting to live in the moment (N=49/59.7%)***

***(Response Frequency by Theme Names and Categories)***

| Categories | Number of patients who expressed DHD（N＝49/59.7%） | |
| --- | --- | --- |
|  | N | ％ |
| Being able to live with optimism, with alleviation of physical pain and peace of mind | 16 | 19.5 |
| Feeling grateful for healthcare workers’ thorough care and trusting them with confidence | 12 | 14.6 |
| Finding hope and meaning in life through family bonds | 12 | 14.6 |
| Feeling responsible for protecting one’s family’s happiness, holding back from death | 11 | 13.4 |
| Increasing one’s will to live through self-encouragement and making an effort | 11 | 13.4 |
| Building relationships with healthcare workers through communication, taking a new look at oneself | 8 | 9.7 |
| Relaxing in a pleasant environment, feeling calm and peaceful | 8 | 9.7 |
| Suppressing anxiety and worries about death, feeling a strong desire to live | 7 | 8.5 |
| Imagining “home” as the place where one belongs, living in the moment | 6 | 7.3 |
